# A micropatterning platform for quantifying interaction kinetics between the T cell receptor and an intracellular binding protein

**DOI:** 10.1038/s41598-019-39865-0

**Published:** 2019-03-01

**Authors:** Viktoria Motsch, Mario Brameshuber, Florian Baumgart, Gerhard J. Schütz, Eva Sevcsik

**Affiliations:** Institute of Applied Physics, TU Wien, Wiedner Hauptstrasse 8-10, 1040 Vienna, Austria

## Abstract

A complete understanding of signaling processes at the plasma membrane depends on a quantitative characterization of the interactions of the involved proteins. Fluorescence recovery after photobleaching (FRAP) is a widely used and convenient technique to obtain kinetic parameters on protein interactions in living cells. FRAP experiments to determine unbinding time constants for proteins at the plasma membrane, however, are often hampered by non-specific contributions to the fluorescence recovery signal. On the example of the interaction between the T cell receptor (TCR) and the Syk kinase ZAP70, we present here an approach based on protein micropatterning that allows the elimination of such non-specific contributions and considerably simplifies analysis of FRAP data. Specifically, detection and reference areas are created within single cells, each being either enriched or depleted in TCR, which permits the isolation of ZAP70-TCR binding in a straight-forward manner. We demonstrate the applicability of our method by comparing it to a conventional FRAP approach.

## Introduction

Interactions between a membrane protein and a cytosolic interaction partner are often involved in the first steps of cell signaling for relaying information across the plasma membrane. Quantification of such interactions in living cells is not a straightforward endeavor; common methods for measuring such intracellular binding kinetics are single molecule imaging methods^1,2^ and fluorescence recovery after photobleaching (FRAP)^2–6^. Since its introduction in 1976^7^, FRAP has become a widely-used technique to probe the dynamic properties of proteins and lipids such as their mobility and localization in different organelles^8,9^. In a typical FRAP experiment, a subset of fluorescently labeled molecules in a defined region of interest (ROI) in a cell is photobleached. The increase of fluorescence in the ROI over time is recorded, yielding information about kinetic rate constants and the fraction of mobile and immobile molecules. Most FRAP studies use confocal microscopy, but due to the small penetration depth of the evanescent field, total internal reflection (TIR) fluorescence microscopes are particularly suited to interrogate processes at the plasma membrane. Confocal as well as TIR-FRAP has been applied to study interaction kinetics between a membrane protein and a fluorescently labeled cytoplasmic protein^2–6,10–12^ but in many cases such experiments are far from straightforward. One complication arises from the fact that typically there are contributions to the fluorescence recovery curve other than the unbinding of bleached and binding of fluorescent protein. For one, the time needed for the protein to diffuse from the cytosol to the binding site –the diffusive recovery –has to be considered in addition to binding^13^. The diffusive recovery may be altered due to unspecific binding of the cytosolic protein directly to the plasma membrane or binding to another membrane protein. Additionally, the membrane protein of interest itself may diffuse into and out of the bleached area. It is difficult if not impossible to properly account for these contributions, particularly considering the lack of a well-defined bleaching geometry, diffusion during the bleach pulse as well as cellular peculiarities such as intracellular local diffusion barriers. Further, fluctuations in brightness due to cell volume changes can influence the fluorescence intensity of the cytosolic protein. Experiments for determining binding kinetics in FRAP experiments are thus often hampered by such non-specific contributions to the signal and great effort has been put into developing experimental protocols as well as analysis approaches to tackle these issues^3,14–17^.

Micropatterning of proteins in the plasma membrane of living cells has been employed by us and others to investigate different protein-protein and protein-lipid interactions^6,18–23^. In this technique, cells are grown on surfaces micropatterned with a specific capture reagent against the protein of interest (bait). By this, the bait gets enriched and immobilized according to the micropatterns directly in the plasma membrane of living cells, leaving the remainder of the cell surface depleted of bait protein. Interaction with a fluorescently labeled interaction partner (prey) can be easily monitored as the appearance of a prey pattern at the position of the bait pattern. A combination of micropatterning and FRAP has been introduced by us previously to probe the binding kinetics of the micropatterned transmembrane protein CD4 and the palmitoylated tyrosine kinase Lck^20^.

Here, we extend and characterize this method for the quantitative analysis of the interaction kinetics of a cytosolic protein and its target protein at the plasma membrane. We exemplify our approach by studying the interaction of ZAP70, a cytoplasmic Syk family kinase, and the T cell receptor (TCR) in Jurkat T cells^2,4,5^. In the course of T cell activation, a stimulating signal initiates a cascade of cellular events that starts with the phosphorylation of tyrosine residues in the immunoreceptor tyrosine-based activation motifs (ITAMs) by Lck. This entails the recruitment of ZAP70 to phosphorylated ITAMs, where it becomes active to phosphorylate downstream targets resulting in a functional T cell response which involves an increase of intracellular calcium levels, cytokine release, proliferation and differentiation^24^. *In vivo*, T cell activation is initiated as a peptide presented by the major histocompatibility complex on antigen-presenting cells is recognized by the cognate TCR, however, surface-immobilized antibodies against a TCR subunit can elicit a similar response^25,26^. We show that protein micropatterning allows the isolation of the specific binding of ZAP70 to the TCR from other contributions to the fluorescence recovery curve in activated T cells thus eliminating sources of error and considerably simplifying analysis.

## Results

### Jurkat T cell activation on OKT3 micropatterns

**Figure 1:**
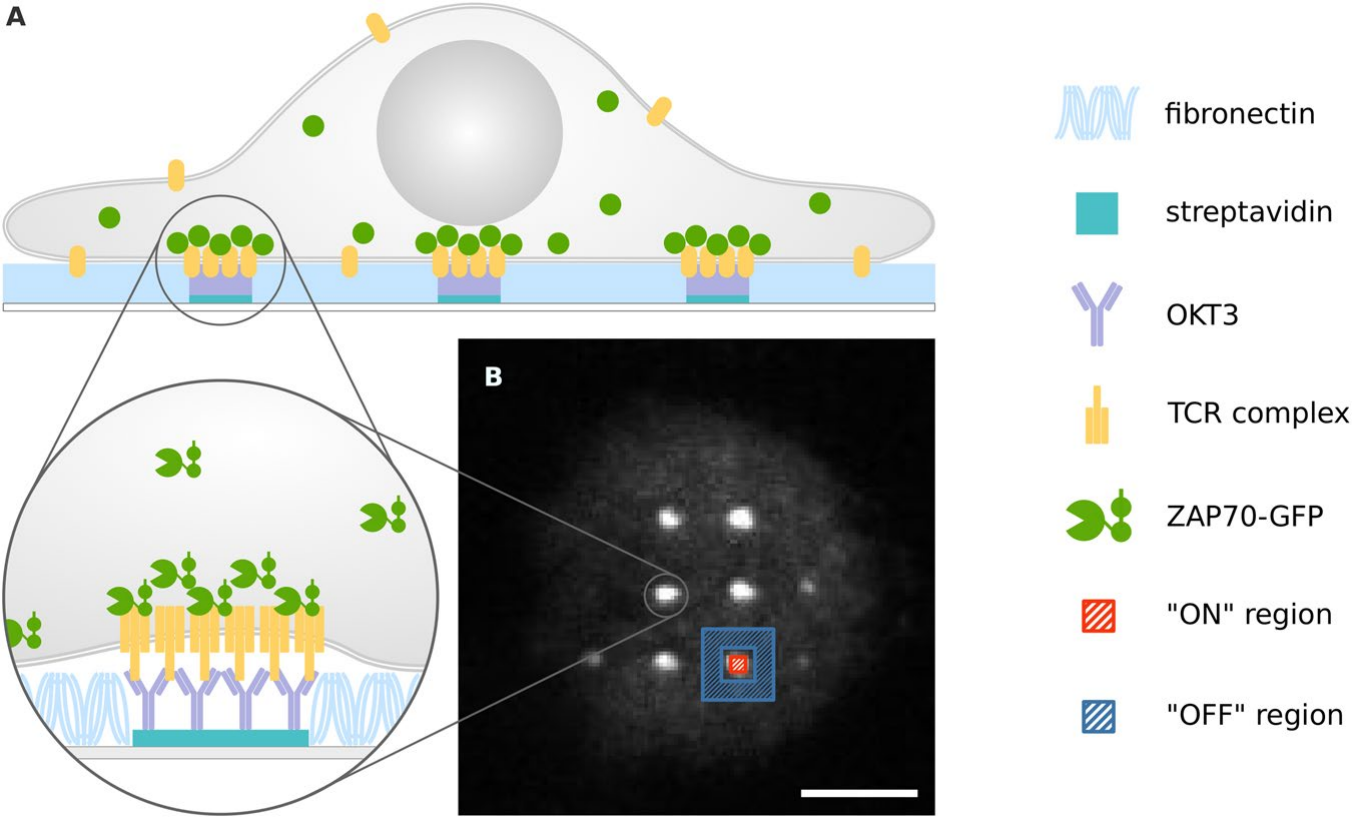
Jurkat T cell on patterned OKT3 antibody surface. (**A**) OKT3 antibody patterns are used to enrich and immobilize the TCR at specific sites at the plasma membrane of living Jurkat T cells. ZAP70-GFP is recruited to these sites. (**B**) Typical micropatterned cell showing enrichment of ZAP70-GFP at OKT3/TCR sites. For analysis, selection masks for TCR-enriched (“ON” -red) and -depleted (“OFF” -blue) areas were generated. Scale bar is 5 µm.

Microstructured surfaces featuring patterns of an activating antibody against the CD3ε-subunit of the TCR complex (OKT3) were used to capture the TCR at defined sites in living Jurkat T cells expressing ZAP70-GFP. Surfaces were created following a protocol described previously^27^; interspaces between 1µm-sized OKT3-enriched dots were filled with fibronectin to promote cell adhesion. Upon surface contact, Jurkat T cells seeded onto patterned surfaces showed fast activation, concomitant with recruitment of ZAP70-GFP to OKT3-containing sites (Figs 1, S1).

**Figure 2:**
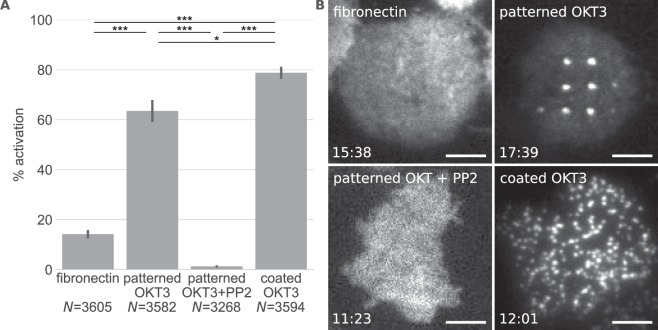
Jurkat T cell activation on the different surfaces. (**A**) Mean percentage of activated cells measured via the increase of intracellular Ca^2+^ levels (*P < 0.05 and ***P < 0.005 (two-tailed unpaired t-test)). Error bars show the SEM in 3 or more different experiments. *N* indicates the total number of measured cells. (**B**) Representative images of ZAP70-GFP distribution in cells on the different surfaces. The timepoint after cell seeding is shown in the bottom left corner. Scale bar is 5 µm.

Jurkat T cell activation was assessed by measuring intracellular calcium levels using the ratiometric calcium-sensitive dye Fura-2, AM. 64% of cells activated within 5 minutes after seeding, which was slightly less than on surfaces homogeneously coated with OKT3 (Fig. 2A). Reduced activation may originate from the lower overall OKT3 density on patterned surfaces since ~90% of the area does not contain OKT3. The local OKT3 density in “ON” areas was similar to surfaces fully coated with OKT3 (Fig. S2). On fibronectin-coated surfaces ~15% of cells activated, similar to what was described previously^28^. Figure 2B shows images of the ZAP70-GFP distribution in representative cells on the different surfaces used in this study. On surfaces homogeneously coated with OKT3, microcluster formation was observed over the whole cell area while on patterned surfaces ZAP70-GFP was enriched at OKT3/TCR sites. The specificity of the enrichment of ZAP70-GFP in CD3 “ON” areas was confirmed by using surfaces patterned with the CD3ε antibody clone MEM-57, which also resulted in ZAP70-GFP patterning, and a non-specific antibody, where no ZAP70-GFP pattern formation was observed (Fig. S3). To further ascertain that the appearance of patterns was a consequence of ZAP70-GFP recruitment to the TCR in the course of T cell activation, cells were treated with the Src kinase inhibitor PP2, which has been shown to block ZAP70 recruitment to the activated TCR on anti-CD3 substrates^4,29,30^. Indeed, T cell activation as well as ZAP70-GFP pattern formation was abolished in the presence of 40 µM PP2 (Fig. 2).

### Complex recovery behavior of ZAP70-GFP in non-activated Jurkat T cells

During a typical FRAP experiment, fluorescent molecules are bleached in a defined region of interest within the cell by a short and powerful laser pulse, and the recovery of fluorescence in the region is monitored by time-lapse microscopy. Before determining ZAP70/TCR binding kinetics in patterned cells, we established our FRAP protocol by measuring the ZAP70-GFP recovery behavior in non-activated cells. For this, cells were seeded onto fibronectin-coated surfaces, allowed to attach and subjected to an intense bleach pulse (for details see Methods section). By using a logarithmically timed illumination protocol we were able to effectively capture the fast recovery of ZAP70-GFP from the cytosol but minimize photobleaching at later time points.

Measured recovery curves were normalized and corrected for bleaching during acquisition and depletion caused by the bleach pulse as described in the Methods section. Although the penetration depth of the TIRF field is limited (~100–200 nm), the bleach pulse causes a significant depletion of the cytosolic pool of ZAP70-GFP; by application of an alternative FRAP protocol we determined this depletion to be ~12% (Fig. S4).

**Figure 3:**
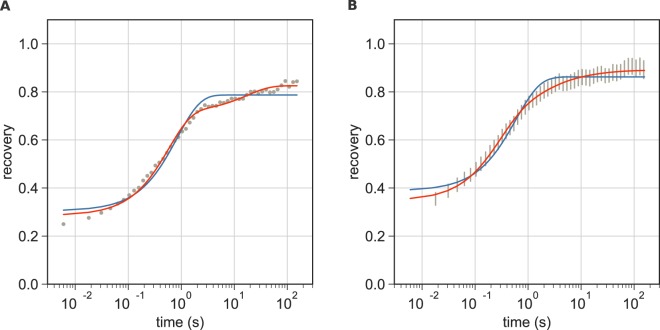
FRAP on non-activated Jurkat T cells. (**A**) A representative FRAP curve of ZAP70-GFP measured in a cell seeded onto a fibronectin-coated surface. A bi-exponential fit (Eq. 2, shown in red) yielded τ_1_ = 0.57 s, τ_2_ = 17.4 s, *f*_*m*_ = 0.83, *b* = 0.29 and *c* = 0.80. A mono-exponential fit (Eq. 1) is shown in blue as a reference. (**B**) Averaged FRAP curves of ZAP70-GFP measured in cells on a fibronectin-coated surface. Data are from three independent experiments with 17 different cells. The 95% confidence interval is shown. Mean values ± SE from cells fitted individually with Eq. 2 were: τ_1_ = 0.29 ± 0.03 s, τ_2_ = 12.6 ± 3.9 s, *f*_*m*_ = 0.89 ± 0.02, *b* = 0.35 ± 0.01 and *c* = 0.67 ± 0.03. The average of all fit curves is shown in red.

The recovery behavior of ZAP70-GFP in cells on fibronectin-coated coverslips showed substantial deviations from the monophasic recovery behavior expected for a protein diffusing in the cytosol with a more slowly recovering component substantially contributing to the recovery curve. FRAP curves of a representative cell as well as pooled FRAP curves of all cells measured are shown in Fig. 3. As an approximation, we fitted our data with an empirical model consisting of one (Eq. 1) or a sum of two exponential functions (Eq. 2).

1 $$F=({f}_{m}-b)\,{e}^{-t/\tau }+b$$

2 $$F=({f}_{m}-b)[1-c{e}^{-t/{\tau }_{1}}-(1-c){e}^{-t/{\tau }_{2}})]+b$$

Here, τ, τ_1_ and τ_2_ are the exponential time constants and *f*_*m*_ is the mobile fraction. *b* is a factor accounting for incomplete bleaching, ZAP70-GFP recovery from the cytosol during the 500 ms bleach pulse and before the first recovery image was taken at t = 6 ms. *c* is the fraction of the first component in the bi-exponential fit.

Several explanations for the origin of the more slowly recovering fraction of ZAP70-GFP come to mind. First, it could be a consequence of cell activation: Ca^2+^ measurements showed that a small percentage of cells (~15%) is activated even under *per se* non-activating conditions. This explanation is unlikely, however, since the slow component was observed not only in a subset but in every measured cell. Second, it is conceivable that ZAP70-GFP experiences transient trapping in membrane ruffles or within the cortical actin or microtubule network. Size-dependent retardation of cytoplasmic molecules by such a ‘molecular sieving’ effect has been proposed previously^17^. Third, the 500 ms bleach-pulse will create a gradient in the bleached cytosolic pool of ZAP70-GFP resulting in a prolonged diffusional recovery^3^. Finally, ZAP70 could transiently bind to one or more unknown membrane proteins or directly to the plasma membrane via its SH2 domain^31^; this membrane-associated fraction would recover more slowly. Application of the same FRAP protocol to Jurkat cells expressing cytosolic GFP also yielded biphasic recovery curves (Fig. S5). Most fit parameters were quantitatively similar for GFP and ZAP70-GFP. Differences were found in the mobile fraction (~11% of ZAP70-GFP did not recover, while cytosolic GFP fully recovered) and the slow recovery rate τ_2_, which was smaller for ZAP70-GFP and showed a larger cell-to-cell variability. Taken together, this suggests that the complex recovery behavior in non-activated cells at least partly arises from cellular structures and/or diffusion during the bleach pulse with possible contributions from a membrane-bound fraction of ZAP70. Interestingly, Loerke *et al*. have described similar diffusional recovery from the cytosol with a slow late component for clathrin light and heavy chains^3^.

### Micropatterning to specifically probe ZAP70 -TCR binding kinetics

**Figure 4:**
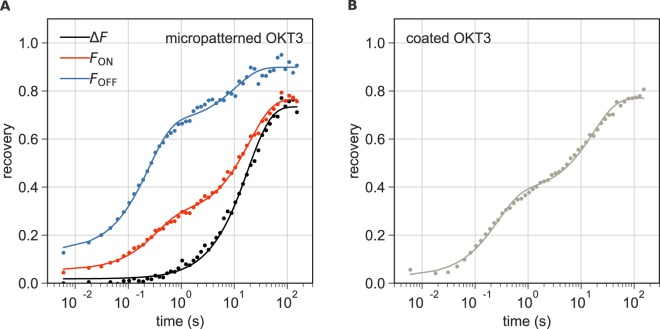
ZAP70-GFP recovery in cells on micropatterned and coated OKT3 surfaces. (**A**) Recovery of fluorescence over time in “ON” and “OFF” areas in a representative micropatterned cell is shown in red and blue, respectively. Fitting with Eq. 2 yielded τ_1_ = 0.2 s, τ_2_ = 10.5 s, *f*_*m*_ = 0.90, *b* = 0.14 and *c* = 0.7 for the “OFF” curve and τ_1_ = 0.3 s, τ_2_ = 19.4 s, *f*_*m*_ = 0.77, *b* = 0.05 and *c* = 0.32 for the “ON” curve. The relative recovery of fluorescence ΔF over time is shown in black and was fit with Eq. 1 yielding τ = 18.1 s, *f*_*m*_ = 0.73, *b* = 0.02. **(B)** Recovery of fluorescence over time in a representative cell on a homogeneously coated OKT3 surface fitted with Eq. 2 yielding τ_1_ = 0.26 s, τ_2_ = 17.0 s, *f*_*m*_ = 0.77, *b* = 0.03 and *c* = 0.47.

In activated Jurkat T cells, the recovery behavior of ZAP70-GFP is expected to contain all contributions present in non-activated cells, but with at least one additional component reflecting its dissociation from the TCR. Figure 4 shows representative recovery curves for activated cells on micropatterned and on homogeneously coated OKT3 surfaces. As an empirical approximation, we again used a bi-exponential fit (Eq. 2) for recovery curves in “ON” and “OFF” areas as well as for those recorded in cells on homogeneously coated OKT3 surfaces. The recovery curves in “OFF” regions (Fig. 4A, blue) feature a slow late component that is even more pronounced than in non-activated cells. In this respect it is interesting to note that Katz *et al*. recently proposed that ZAP70 is released from the TCR into the plasma membrane via binding to an unknown partner in early TCR signaling^2^. This was suggested to be a mechanism of spreading the signal to downstream molecules such as LAT. It is thus conceivable that an additional fraction of ZAP70 diffuses at the plasma membrane of T cells upon activation and contributes to the recovery curve.

The analysis of ZAP70 recovery curves in activated Jurkat T cells in “ON” areas or on homogeneously coated OKT3 surfaces is even more complicated. The key advantage of the micropatterned surfaces in this respect is that contributions specific to the TCR binding can be isolated in a straight-forward manner: in OKT3-free “OFF” areas of the micropatterned cells, fluorescence recovery of ZAP70-GFP is governed by cytosolic diffusion plus other non-specific contributions (Fig. 4A, blue). They can serve as reference areas to correct the fluorescence recovery in the detection (OKT3 “ON”) areas (Fig. 4A, red): the relative recovery of fluorescence in the “ON” areas compared to “OFF” areas, ${\rm{\Delta }}F={F}_{ON}-{F}_{OFF}$, only contains the contribution of ZAP70-GFP binding to the TCR (Fig. 4A, black), follows an exponential time course and can thus be fit with Eq. 1.

**Figure 5:**
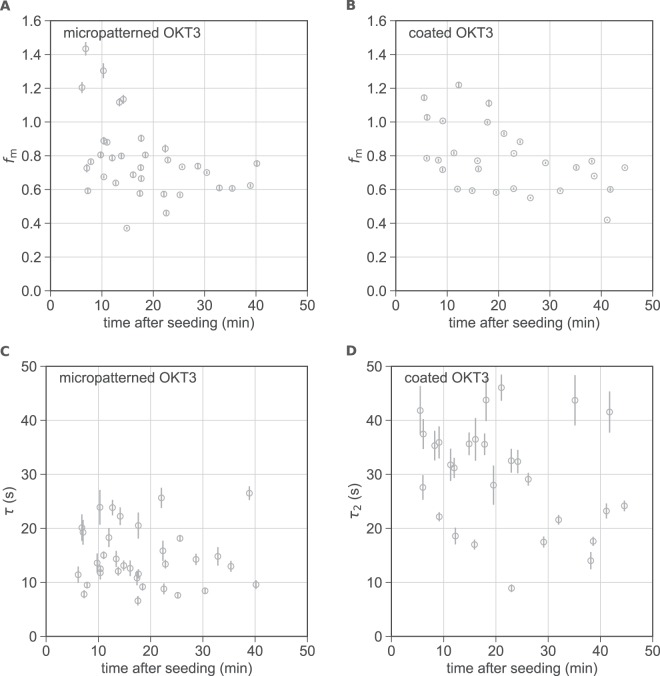
Comparison of FRAP parameters for cells on micropatterned and homogeneously coated OKT3 surfaces at 22 °C. The fraction of mobile ZAP70-GFP *f*_*m*_ (**A,B**) and the unbinding time constants τ and τ_2_ (**C,D**) are plotted over the time after seeding for cells on micropatterned (*N* = 34) (**A,C**) and homogeneously coated (*N* = 28) (**B,D**) OKT3 surfaces. Error bars show the SE of the fit.

Next, we compared the key FRAP parameters (recovered fraction and unbinding time constant) measured using the micropatterning approach with those extracted from cells on homogeneously coated OKT3 surfaces using Eq. 2. Previous studies had reported a time-dependence of the mobile fraction of ZAP70^2,4^; furthermore, some cell-to-cell variability was to be expected due to different expression levels of the T cell receptor and proteins involved in the signaling cascade such as Lck and CD45. We therefore chose to fit recovery curves individually and plotted the key FRAP parameters as a function of the measurement time point (Fig. 5). A listing of all extracted averaged parameters is given in Table 1; the FRAP parameters τ_1_, *b* and *c* plotted as a function of time can be found in Fig. S6. In the following we compare data and fit results obtained from cells on micropatterned and homogeneously coated OKT3 surfaces:

**Table 1:** Parameters extracted from FRAP curves. Cells seeded onto micropatterned and homogeneously coated OKT3 surfaces were fit individually with Eq. 1 and Eq. 2, respectively.

T	min after seeding		τ_1_(s)	τ_2_/τ(s)	*f*_*m*_	*b*	*c*	*N*
**22 °C**	**5–45**	patterned OKT3	—	14.6 ± 1.0	0.78 ± 0.04	0.12 ± 0.02	—	34
		coated OKT3	1.0 ± 0.1	29.7 ± 1.9	0.78 ± 0.04	0.10 ± 0.02	0.34 ± 0.02	28
**37 °C**	**5–15**	patterned OKT3	—	6.7 ± 1.1	0.68 ± 0.03	0.07 ± 0.01	—	8
		coated OKT3	1.6 ± 0.3	15.6 ± 3.7	0.76 ± 0.04	0.21 ± 0.05	0.33 ± 0.07	7
	**25–45**	patterned OKT3	—	4.7 ± 0.90	0.57 ± 0.04	0.05 ± 0.01	—	8
		coated OKT3	1.2 ± 0.2	20.7 ± 5.3	0.57 ± 0.05	0.17 ± 0.04	0.43 ± 0.05	8

Mean fit values ± SEM are listed for τ_1_, τ_2_, *f*_*m*_*, b* and *c*. For cells measured at 22 °C, all cells shown in Fig. 5 were included. For data recorded at 37 °C, cells were grouped according to the timepoints of their measurement (5–15 minutes and 25–45 minutes after seeding). *N* indicates the number of cells in the time group. Data were recorded in at least 2 independent experiments.

### Mobile fraction

**Figure 6:**
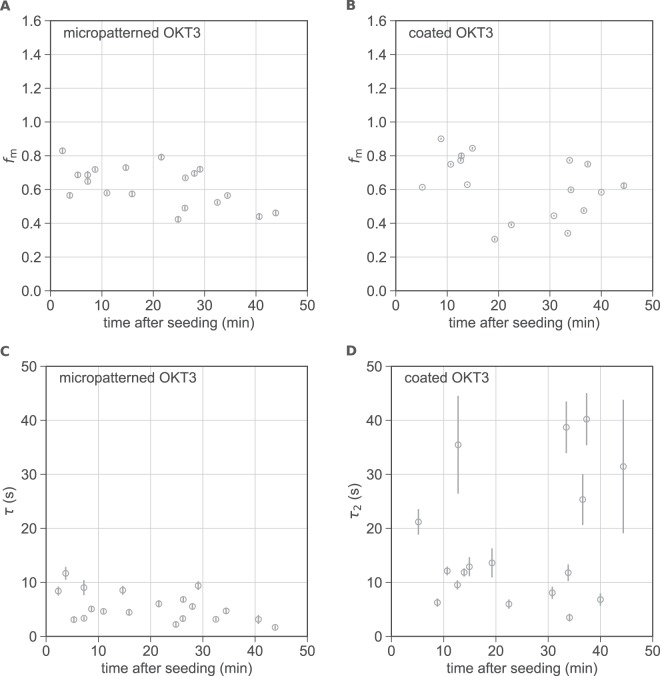
Comparison of FRAP parameters for cells on micropatterned and homogeneously coated OKT3 surfaces at 37 °C. The fraction of mobile ZAP70-GFP (**A,B**) and the unbinding time constants τ and τ_2_ (**C,D**) are plotted over the time after seeding for cells on micropatterned (*N* = 19) (**A,C**) and homogeneously coated (*N* = 17) (**B,D**) OKT3 surfaces. Error bars show the SE of the fit.

As shown in Table 1 and Fig. 5, both approaches yield a mobile fraction *f*_*m*_ of 78% in cells measured at 22 °C. Note that here, particularly at early timepoints, some cells exhibit a recovery of fluorescence intensity above 100%. In these cases, we observed one or more dots or microclusters appearing rather dim in the prebleach image and increasing in brightness during the recovery process. Most likely, this reflects new recruitment of ZAP70-GFP to sites of immobilized TCR (on top of exchange of previously bound ZAP70) or additional accumulation of TCR at such sites. Note that not all cells make surface contact at the same time, thus the time axis may contain some uncertainty. Apart from this effect, we did not observe a marked correlation of the mobile fraction with the time after cell seeding. Previous studies conducted at 37 °C have suggested that in late signaling (after 30 min) a conformation of ZAP70 becomes dominant that is more stably associated with the TCR and does not recover on the timescale of 2 minutes. The mobile ZAP70 fraction in the first 10 minutes after cell plating was reported to range from ~65 to 85% and decreased to ~30% after 30 minutes^2,4^. At 22 °C, T cell activation is slowed down in comparison and it is likely that late signaling stages are not reached within the 45 minutes of our experiments. To address this point in more detail, we repeated our experiments at 37 °C. Indeed, we found a pronounced time dependence of the mobile ZAP70-GFP fraction in cells on patterned and homogeneously coated OKT3 surfaces (Fig. 6, Table 1). While in the first 15 minutes after seeding, the fluorescence recovery was 68 and 76%, on patterned and coated OKT3 surfaces, respectively, these values both decreased to 57% after 25 minutes. A quantitative comparison of values with previously published data is difficult due to the different treatment of the offset of the recovery curves (taken here into account as *b* in Eqs 1 and 2, but not in earlier work^2,4^). The FRAP parameters τ_1_, *b* and *c* plotted as a function of time for cells measured at 37 °C can be found in Fig. S7.

### Interaction times

ZAP70-GFP unbinding time constants extracted from patterned cells at 22 °C were not dependent on the time after seeding (Fig. 5C,D); at 37 °C a minor dependence was observed. As expected, time constants were lower at 37 °C than at 22 °C (by a factor of 2–2.5). In contrast to mobile fractions, ZAP70-GFP unbinding time constants differed between cells on micropatterned and those on OKT3-coated surfaces (Table 1, Figs 5C,D, 6C,D). In general, time constants extracted from cells on micropatterned OKT3 surfaces were lower and showed less variability than on homogeneously coated OKT3 surfaces. One aspect contributing to the heterogeneity observed for cells on coated OKT3 surfaces is that fitting a sum of exponentials, especially to rather noisy data such as single cell FRAP curves, is known to be an ill-conditioned problem and thus prone to yield erroneous fit parameters^32^. This is further complicated by the fact that an exponential function does not correctly describe the fast recovery from the cytosol.

Most importantly, it is likely that non-specific contributions compromise the ZAP70-GFP recovery data recorded for cells on homogeneously coated OKT3 surfaces, leading to an overestimation of the unbinding time constants. Indeed, this effect was previously observed by Loerke *et al*. in TIR-FRAP measurements of clathrin heavy and light chain exchange in clathrin-coated pits^3^. The authors found that fluorescence recovery in pits was not exponential, and the recovery outside of pits had a late slow-rising component. The authors then corrected their FRAP data in clathrin-coated pits using an approach similar to ours for micropatterns and the diffusion-corrected traces could be approximated well by a mono-exponential function. Interestingly, their extracted time constants were smaller when compared to those previously reported in the literature by a factor of 2. The authors suggested diffusion artifacts to account for this discrepancy. These findings are surprisingly similar to our observations for ZAP70-GFP: previously, ZAP70-GFP unbinding half-times of ~22 s^2^ and 7.5–10s^4^, corresponding to τ ~ 32 s and τ ~ 11–14 s, have been reported at 37 °C. This is consistent with the τ_2_ values we extracted from cells on homogeneously coated OKT3 surfaces (16–21 s) but higher than the values we determined on patterned OKT3 surfaces at 37 °C (~5–7 s). Apart from unspecific contributions from cytosolic diffusion, a fraction of slowly diffusing membrane-bound ZAP70-GFP could additionally contribute to the recovery curve for cells on coated OKT3 surfaces but would be eliminated in the micropatterning approach.

In conclusion, we show that the combination of protein micropatterning and FRAP is a convenient and simple approach to specifically determine binding kinetics between a cytosolic protein and a transmembrane interaction partner. We validate our method on the example of ZAP70 binding to the TCR and show that non-specific contributions to the fluorescence recovery signal can indeed be removed. Thus, the corrected recovery curves only reflect the interaction times of the two proteins and can be fit with a mono-exponential function in a straightforward manner. In the case of the TCR-ZAP70 interaction, formation of immobile TCR microclusters simplifies FRAP analysis. Our micropatterning approach, however, will also be particularly useful for measuring the interaction of a cytosolic protein and an otherwise mobile membrane protein.

## Methods

### Cells and reagents

The plasmid DNA for hZAP70-eGFP was kindly provided by B. Lillemeier (Salk Institute for Biological Studies, USA) and the plasmid for cytosolic GFP was a gift from H. Stockinger (Medical University of Vienna, Austria). Both constructs were recloned into the retrovirus vector backbone pBMN-Z. Phoenix packaging cells were co-transfected with the vector and pCL-eco using TurboFect™ Transfection Reagent (Invitrogen Life Technologies, USA). After 2 days of virus production, the supernatant was mixed with 10 µg/ml polybrene (Sigma-Aldrich, USA), added to Jurkat E6–1 T cells from the American Type Culture Collection followed by spin infection. Cells were cultured in RPMI 1640 medium supplemented with 10% fetal bovine serum (FBS), 2 mM L-glutamine, 1000 U/ml penicillin–streptomycin (all from Sigma-Aldrich, USA) in a humidified atmosphere at 37 °C and 5% CO_2_. For microscopy, we used an imaging buffer consisting of HBSS (Lonza, Switzerland) supplemented with 2% FBS. For kinase inhibition, cells were incubated in imaging buffer with 40 μM PP2 (Sigma-Aldrich, USA) for 5 min before and during the experiment. Biotinylated monoclonal antibody against CD3ε (clone: OKT3) and biotinylated anti-HA.11 Epitope Tag antibody (clone: 16B12) were purchased from BioLegend (USA). Monoclonal biotinylated anti-CD3ε antibody (clone: MEM-57) was purchased from Sigma-Aldrich (USA).

### Surface preparation

Micropatterned OKT3 surfaces were produced following a previously published protocol^27^. Briefly, polymer stamps featuring circular pillars with a diameter of 1 μm and a center-to-center distance of 3 μm (EV group, Austria) were cleaned with absolute ethanol and dH_2_O, then incubated with 50 μg/ml streptavidin (AppliChem, Germany) in phosphate-buffered saline (PBS, Sigma-Aldrich, USA) for 15 min, rinsed with dH_2_0, and dried in a N_2_ flow. After drying, the stamp was placed on an epoxy-coated coverslip (Schott, Germany), pressed to ensure good contact and incubated for 30 min at 22 °C. After removal of the stamp, a Secure-Seal hybridization chamber (Grace Biolabs, USA) was placed on top of the pattern and 10 μg/ml fibronectin (Sigma-Aldrich, USA) in PBS with 1% bovine serum albumin (BSA, Sigma-Aldrich, USA) was added. After 30 min incubation, the structures were rinsed with PBS and incubated with biotinylated antibody at a concentration of 10 μg/ml in PBS with 1% BSA. After 15 min samples were rinsed thoroughly with PBS. Homogeneously coated OKT3 surfaces were produced by placing a Secure-Seal hybridization chamber onto an epoxy-coated coverslip and adding OKT3 antibody at a concentration of 10 μg/ml in PBS into the chamber. After 15 min samples were rinsed thoroughly with PBS. Fibronectin-coated surfaces were produced by placing a Secure-Seal hybridization chamber on an epoxy-coated coverslip and adding 10 μg/ml fibronectin in PBS into the chamber. After 30 min incubation, samples were rinsed thoroughly with PBS.

### Total internal reflection fluorescence (TIRF) microscopy

TIRF microscopy experiments were performed on a home-built system based on a modified inverted microscope (Zeiss Axiovert 200, Germany) equipped with a 100× oil-immersion objective (Zeiss Apochromat NA1.46, Germany). For FRAP experiments, a 488 nm diode laser (Toptica ibeam-smart, Germany) was used. Emission light was filtered using appropriate filter sets (Chroma, USA) and recorded on an iXon DU 897-DV EM-CCD camera (Andor, Ireland). Total internal reflection fluorescence (TIRF) illumination was achieved by shifting the excitation beam in parallel to the optical axis with a mirror mounted on a motorized movable table. For experiments performed at 37 °C the temperature was maintained by means of an in-house-built incubator box equipped with a heating unit and an objective heater (PeCon, Erbach, Germany). For characterization of patterned and coated OKT3 surfaces, prepared slides were incubated with the Zenon™ Alexa Fluor™ 647 Mouse IgG2a Labeling Kit (Molecular Probes, USA) according to the manufacturer’s protocol and imaged with a 640 nm diode laser (Coherent, Obis, USA). To determine the antibody densities, the mean bulk brightness of the different surfaces was compared. For patterned surfaces, selection masks defining “ON” and “OFF” regions were applied.

### Ratiometric Ca^2+^ Imaging

For ratiometric Ca^2+^ imaging, cells were incubated with 5 μg/ml Fura-2, AM (Thermo Fisher Scientific, USA) in imaging buffer for 20 min at 22 °C and washed twice with imaging buffer. For each experiment, cells were resuspended in imaging buffer at 5 × 10^6^ cells/ml and 50 μl were deposited in a Secure-Seal hybridization chamber, which was mounted on the microscope. Image acquisition was started immediately. Fura-2-AM was excited using a monochromatic light source (Polychrome V, TILL Photonics), coupled to a Zeiss Axiovert 200 M equipped with a 20 × objective (Olympus) and an Andor iXon Ultra. Imaging was performed at 340 nm or 380 nm at an illumination time of 50 ms or 10 ms, respectively. The total recording time was 10 min at 1 Hz. ImageJ was used to generate the ratio images. Cells were segmented and tracked using a sum image of both channels using an in-house Matlab algorithm based on published literature^33^. Cellular positions and tracks were stored and used for intensity extraction based on the ratio image. Intensity traces were normalized to the starting value at time point zero; a cell was considered as activated if a threshold set at 0.4 was exceeded.

### FRAP experimental procedure

All cell measurements were carried out in HBSS supplemented with 2% FBS at 22 °C unless stated otherwise. Experiments were carried out 5 to 45 min after cell seeding. A prebleach image (exposure time: 5 ms) was acquired followed by a bleach pulse of 500 ms duration. Imaging was performed at low laser power (150 W/cm^2^) to minimize photobleaching; bleach pulses were performed at 4000 W/cm^2^. 50 postbleach images were taken with delays between consecutive images being evenly spaced on a logarithmic scale with the first postbleach image taken at 6 ms after the end of the bleach pulse.

Control experiments to determine the amount of photobleaching during image acquisition were performed using the same timing protocol as used for the FRAP experiments and replacing the bleach pulse with a standby time of the same duration. Since the recovery curves of ZAP70-GFP as well as cytosolic GFP contained contributions other than pure cytosolic diffusion of unknown origin, we chose to determine the extent of depletion during the bleach pulse by applying an alternative FRAP protocol. Cells expressing ZAP70-GFP seeded onto fibronectin-coated surfaces were subjected to repetitive 500 ms bleach pulses separated by 1.5 s to isolate the fast recovering component. Images were read out 1 s after the end of each bleach pulse. To determine the extent of depletion, the basal plasma membrane of a cell (excluding cell edges) was selected as a region of interest and the relative change of the background corrected mean pixel intensity values for each region between pre- and post-bleach image was calculated. For each cell, the intensity change caused by the first bleach pulse was discarded and the mean recovering fraction was calculated from the subsequent four bleach pulses. Values were determined separately for cells at 22 °C and 37 °C.

### FRAP analysis

For analysis of FRAP curves on patterned cells, selection masks were created from the pre-bleach image using ImageJ and applied to all postbleach images. For “ON” regions, a rectangular ROI inside the ZAP70-GFP dot was selected; for the corresponding “OFF” region, a rectangular annulus around “ON” regions was selected as shown in Fig. 1. The background corrected mean pixel intensity values for each region, ${F}_{ON}$ and ${F}_{OFF}$, were used for further analysis. Only dots with a minimal contrast of 0.4 were included in the analysis with the contrast being defined as $\frac{{F}_{ON}-{F}_{OFF}}{{F}_{ON}}$. The number of ROIs measured in each cell was strongly dependent on the cell size, with a mean of 6.7 ± 0.51 (SEM) ROIs for all patterned cells. There was no correlation between the number of ROIs and either *f*_*m*_ or *τ*. For cells on homogeneously coated surfaces, the basal plasma membrane of a cell (excluding cell edges) was selected as a region of interest in ImageJ. The following steps in the analysis of FRAP data were implemented using Python (Python Software Foundation). ${\rm{\Delta }}F={F}_{ON}-{F}_{OFF}$ in micropatterned cells was determined separately for each dot of the pattern by subtracting the mean pixel intensity value of each “OFF” area region from the mean pixel intensity value of the respective “ON” region. All dots in a cell were combined to generate a FRAP curve. Then, FRAP curves were normalized to the pre-bleach value of the respective pulse train. To correct for photobleaching during image acquisition, each datapoint of a FRAP curve was divided by the respective datapoint of the control curve (recorded as described in the previous section). Further, to correct for depletion of the fluorescent ZAP70-GFP pool by the bleach pulse, each postbleach datapoint of a FRAP curve was divided by the determined depletion correction value of 0.88 and 0.85 for data collected at 22 °C and 37 °C, respectively. We used the scipy.optimize.curve_fit function for performing non-linear least squares to fit Eq. 1 and Eq. 2 to the data. A Trust Region Reflective algorithm was used for optimization. FRAP data were fit separately for each cell.

## Supplementary information

Supplementary Information
