# Supplementary material for: A micropatterning platform for quantifying interaction kinetics between the T cell receptor and an intracellular binding protein
Source: Sci Rep. 2019 Mar 1;9:3288. doi: 10.1038/s41598-019-39865-0 (PMC6397226; doi:10.1038/s41598-019-39865-0)
Supplement: Supplementary file 1 — Supplementary Information [file 41598_2019_39865_MOESM1_ESM.pdf]

A micropatterning platform for quantifying interaction kinetics between the T cell receptor and an intracellular binding protein

Viktoria Motsch, Mario Brameshuber, Florian Baumgart, Gerhard J. Schütz, and Eva Sevcsik

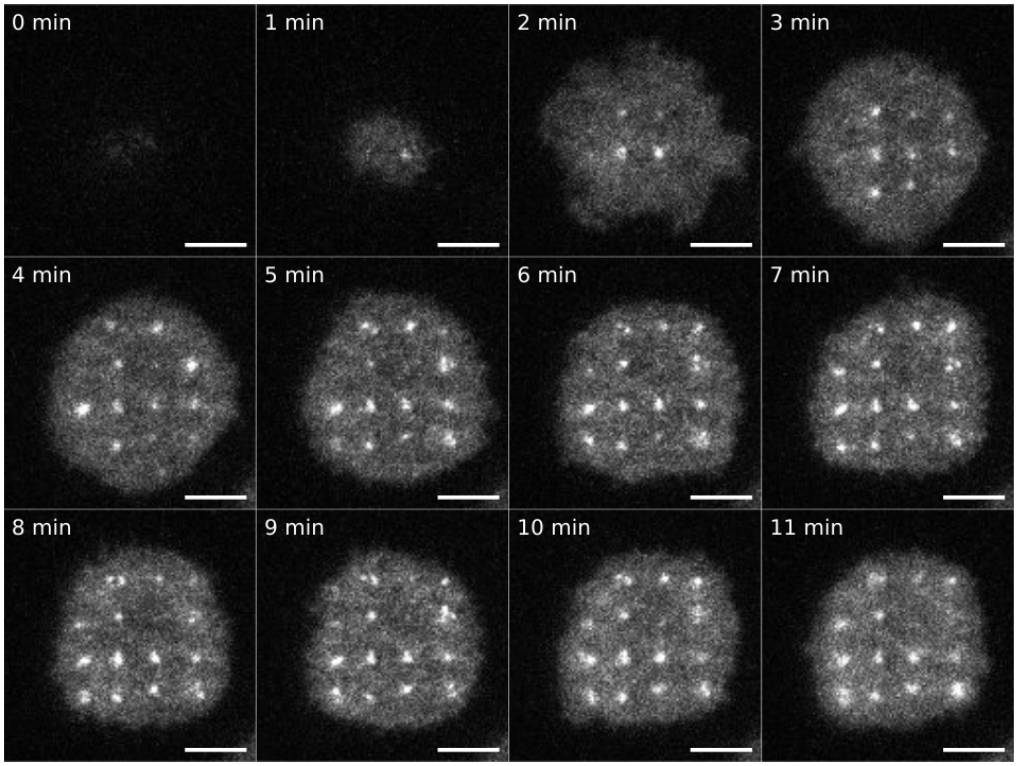

Figure S1. Time course of ZAP70-GFP recruitment to OKT3 sites. Cells were seeded onto a micropatterned OKT3 surface at t = 0 min. Cell attachment and formation of ZAP70-GFP patterns over time is shown for a representative cell. Scale bar is 5 µm.

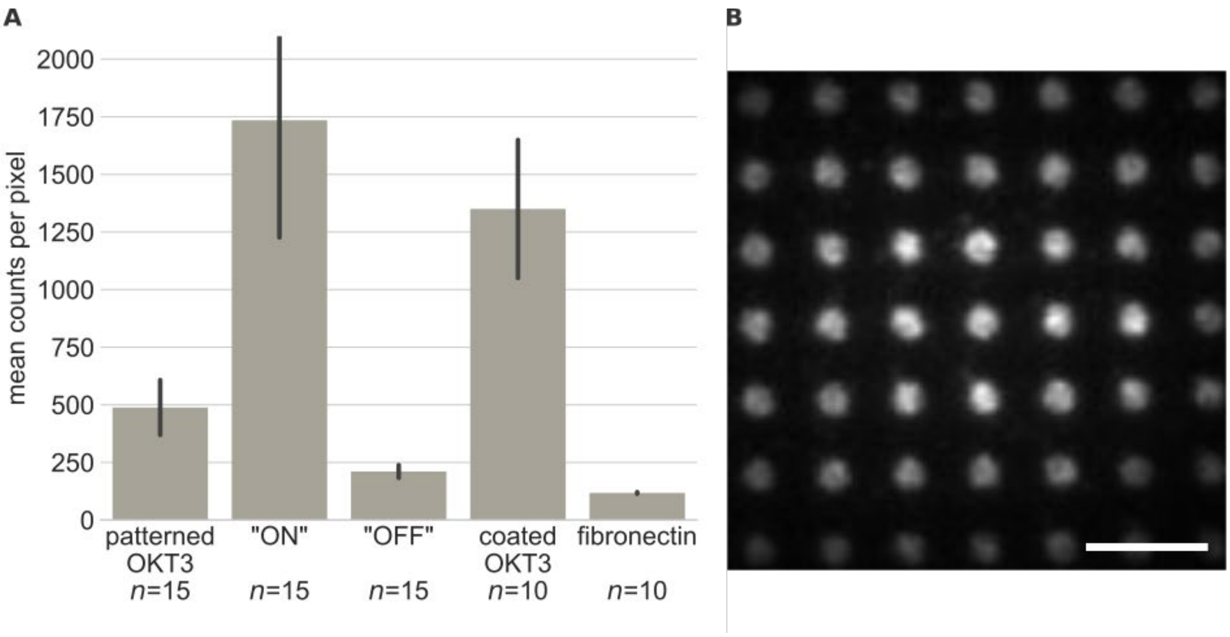

Figure S2. Relative OKT3 antibody densities on patterned and homogeneously coated OKT3 surfaces. (A) OKT3 antibodies were labeled with Zenon™ Alexa Fluor™ 647 Mouse IgG2a Fab fragments. Relative antibody densities are given by the fluorescence intensities. Data are from three independent experiments (average ± SEM); the number of measured areas is indicated as *n*. **(B)** Image of a patterned OKT3 surface labeled with Zenon™ Fab fragments. Scale bar is 5 µm.

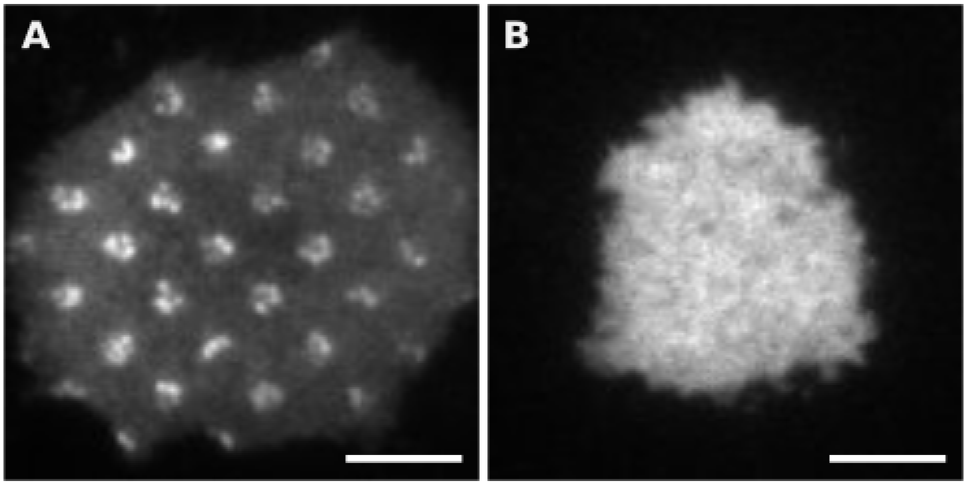

Fig S3: Specific ZAP70-GFP recruitment to aCD3 "ON" areas. Cells expressing ZAP70-GFP were seeded onto micropatterned surfaces featuring antibodies against **(A)** the CD3ε epitope MEM-57 and **(B)** an HA-tag and allowed to adhere for 10 minutes before an image of the ZAP70-GFP distribution was recorded.

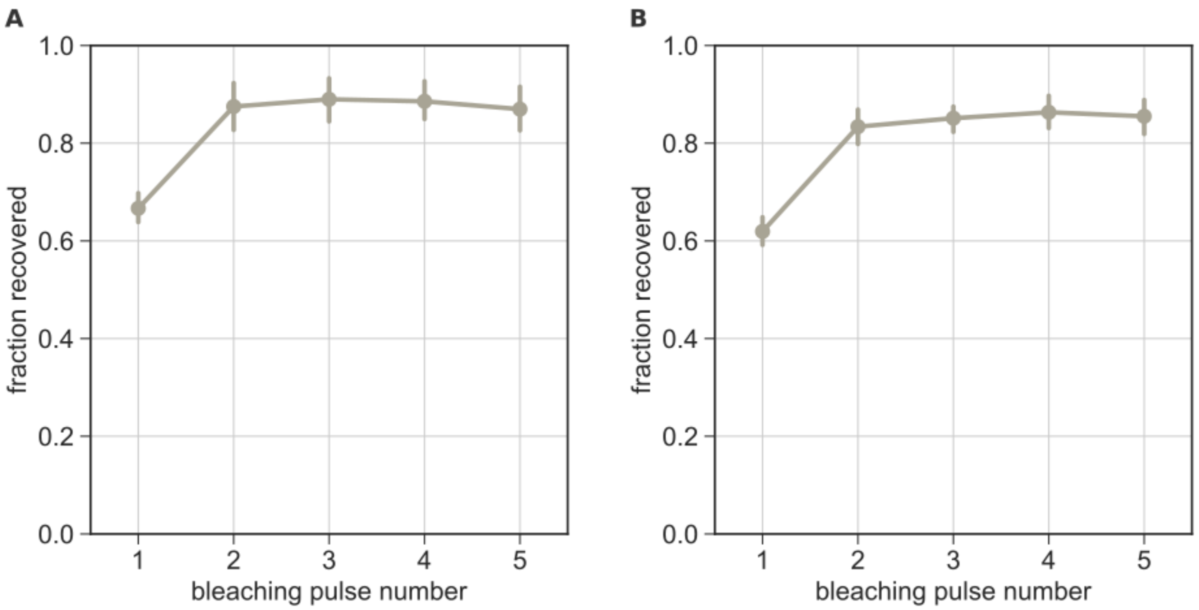

Figure S4. Recovering fraction of Zap70-GFP after repetitive bleach pulses. Images were recorded 1s after the end of a 500ms bleach pulse and normalized to the corresponding pre-bleach image. Data are from two independent experiments with 50 **(A)** and 21 **(B)** different cells (average + 95% confidence interval). The short time interval between bleach pulses does not allow for the recovery of the slower fraction of ZAP70-GFP. The recovering fraction increases with successive FRAP experiments to level out at ~ 88% **(A)** and ~85% **(B)**, indicating that the depletion of the cytosolic pool of ZAP70-GFP caused by the bleach pulse amounts to ~12% and ~15% at 22°C and 37°C, respectively.

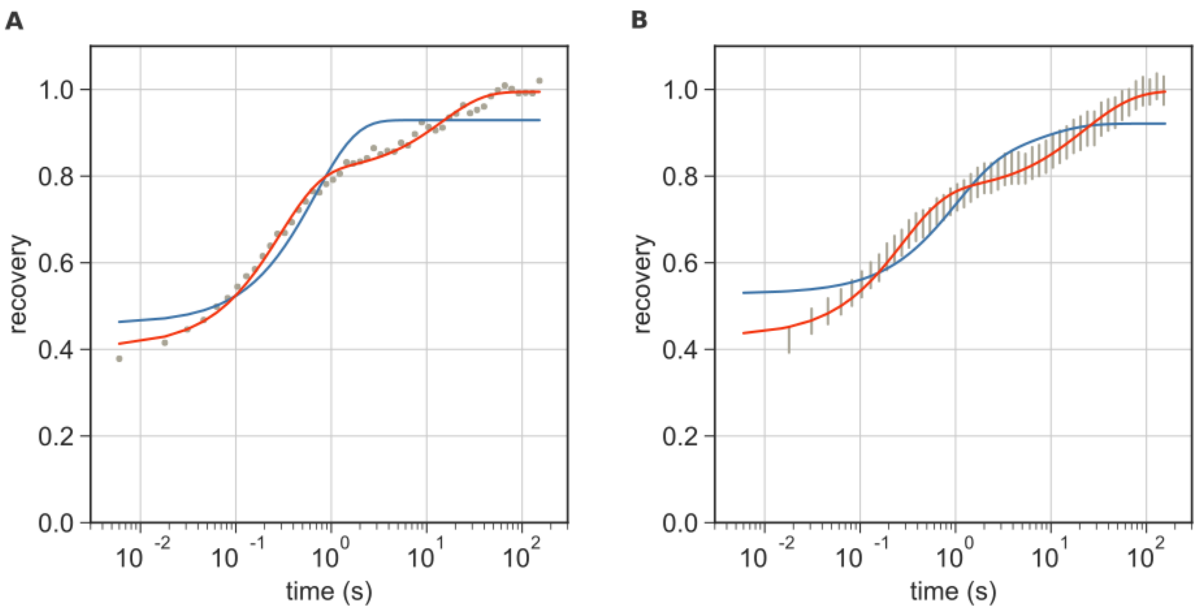

Figure S5. FRAP in cells expressing cytosolic GFP on a fibronectin-coated surface. (A) A representative FRAP curve of cytosolic GFP measured in a cell on a fibronectin-coated surface. A mono-exponential fit (Eq. 1) is shown in blue. A bi-exponential fit (Eq. 2, shown in red) yielded τ_1 = 0.29s, τ_2 = 14.46s, f_m = 0.99, *b* = 0.40 and *c*= 0.68. **(B)** Averaged FRAP curves of cytosolic GFP measured in cells on a fibronectin-coated surface. Data are from three independent experiments with 29 different cells. The 95% confidence interval is shown. Mean values ± SE from cells expressing cytosolic GFP fitted individually with Eq. 2 were: τ_1 = 0.28 ± 0.02s, τ_2 = 25.4 ± 2.4s, f_m = 1.00 ± 0.01, *b* = 0.43 ± 0.01 and *c*= 0.59 ± 0.02. The average of all fit curves is shown in red.

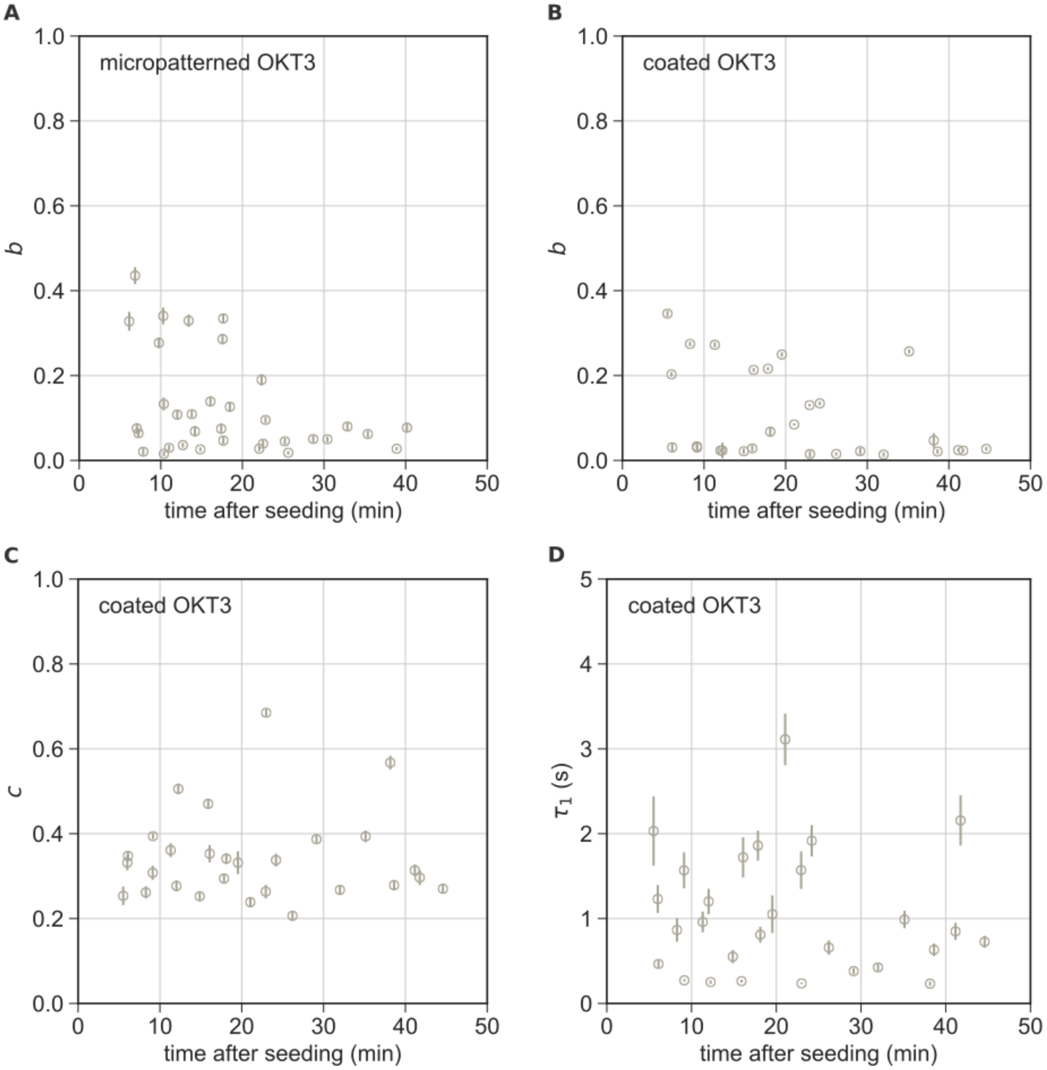

Figure S6. Comparison of FRAP parameters for cells on micropatterned and coated OKT3 surfaces at 22°C. The fitting parameters *b, c* and τ_1 are plotted over time after seeding for cells on micropatterned (N=34, 3 independent experiments) **(A)** and homogeneously coated (N=28, 4 independent experiments) **(B-D)** OKT3 surfaces. Error bars show the SE of the fit.

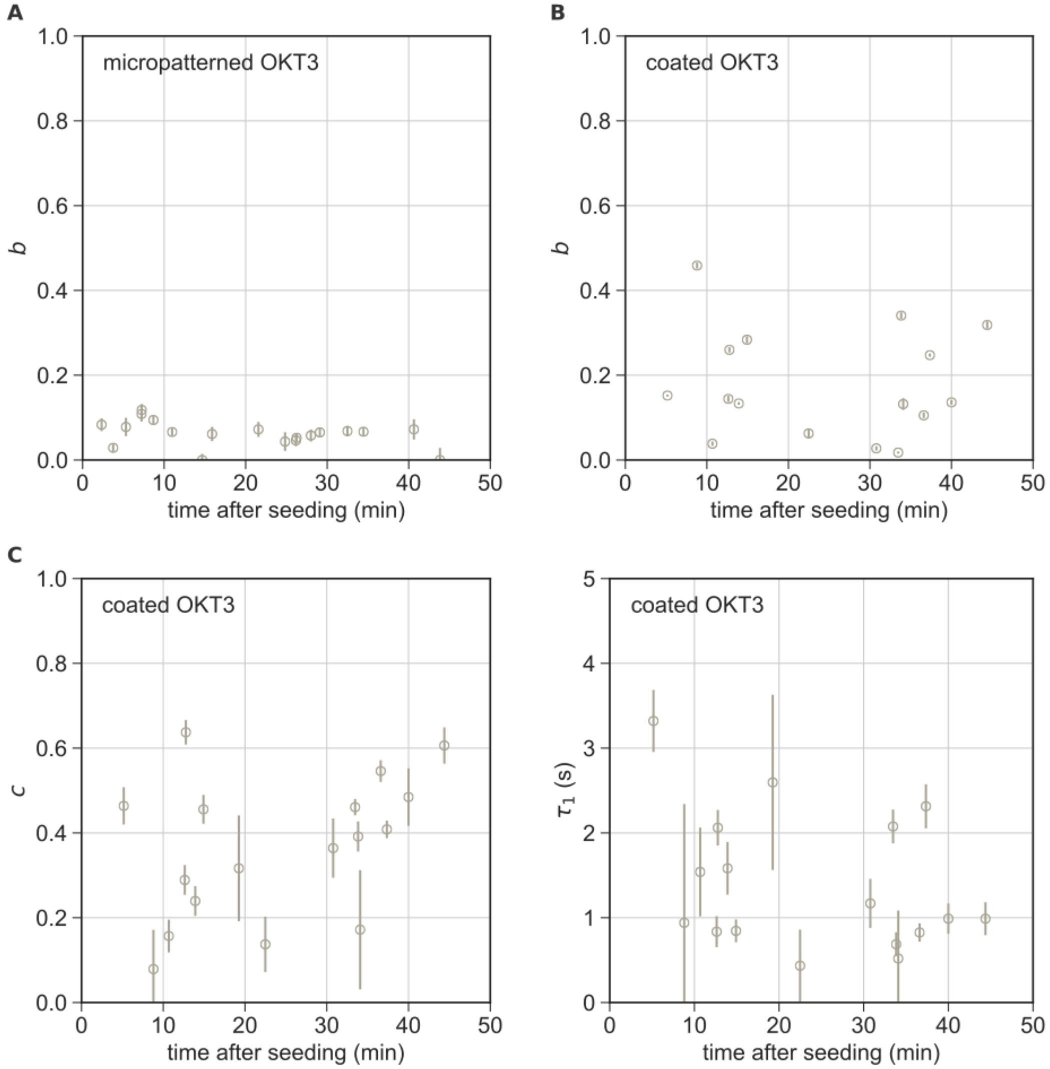

Figure S7. Comparison of FRAP parameters for cells on micropatterned and coated OKT3 surfaces at 37°C. The fitting parameters *b, c* and τ_1 are plotted over time after seeding for cells on micropatterned (N=19, 2 independent experiments) **(A)** and homogeneously coated (N=17, 2 independent experiments) **(B-D)** OKT3 surfaces. Error bars show the SE of the fit.
